# Exploring new dimensions in cardiovascular flow and motion: application of Bloch NMR flow equations, Bessel and spherical harmonic functions

**DOI:** 10.1186/1532-429X-14-S1-W28

**Published:** 2012-02-01

**Authors:** Bamidele O Awojoyogbe

**Affiliations:** 1 Department of Physics, Federal University of Technology Minna, Minna, Nigeria

## Summary

Biological tissues are complex system that contains a variety of liquid components, macromolecules and ions. They are highly heterogeneous media that consist of various compartments and barriers of different diffusivities. In terms of its cyto-histologic architecture, a tissue can be regarded as a porous structure made up of a set of more or less connected compartments in a network - like arrangement. Since molecular motion of water is significantly affected by macromolecules, the variation in the relaxation times between tissues is attributed to the effect of macromolecular interaction. The movement of water molecules during diffusion-driven random displacement is restricted by compartmental boundaries and other molecular obstacle in such a way that the actual diffusion distance is reduced, compare with what is expected in unrestricted diffusion. In this study, based on the new NMR diffusion equation, Bessel functions and the spherical harmonic functions, we present an analysis of diffusing blood component by monitoring its NMR signal emitted at its unique resonant frequency. This view can be very important in the study of pathologies such as sickle cell anemia and finding a treatment to the genetic disorder. This is because, we may be able to visualize blood vessels pores that are blocked by the sickle cell and find out the factor that makes the erythrocytes rigid. It is expected that the diamagnetic property of the oxyhemoglobin that makes it difficult to deliver the oxygen on it would be very useful here. Hence, a very slowly decaying signal or non - decaying signal could be the indication of some form of problem with the vessel.

## Background

Analytical solutions to the Bloch NMR flow equations can provide a straightforward means of obtaining fundamental and accurate information on the translational motion of nuclear spins for dynamic analysis of fluid flow in biological and non biological systems. However, the interpretation of the data is complicated by the effects of restricting geometries. The mathematics required to model diffusion coefficient for diffusion within restricting geometries especially for medical imaging has been very difficult because analytical solutions to the Bloch NMR flow equations are generally considered not possible. Therefore, numerical and approximate solutions are the only options available for the analysis of special arrangement of porous media such as biological and liquid crystals systems. In this contribution, we have presented the analytical solutions to the inhomogeneous second order differential NMR diffusion equation in cylindrical and spherical coordinates for theoretical, computational and possible experimental studies of fluids in restricted geometries.

## Methods

We derive analytical expressions for the NMR transverse magnetization and diffusion coefficient which can be detected by the recovery unit in the MRI scanner based on the Bloch NMR flow equations with the assumption that resonance condition exists at Larmor frequency.

## Results

The oxygen has an equal probability of being anywhere in the oxyhemoglobin molecular box. Similarly, the relative difference in energy between levels becomes too small to measure, so the energy that the oxygen can have appears continuous rather than quantized when the quantum numbers become large. Both position and energy can be described by classical mechanics at large quantum numbers.

## Conclusions

We have presented a model of diffusing blood flow component by monitoring it’s NMR signal quantum mechanically. The wave properties and boundary conditions imposed on the modeled system lead to quantized energies of the oxygen molecules. As the cells stretch into thin shapes so as to pass through the tiniest blood vessels and thus sustain every part of the body, the size of haemoglobin molecular box changes and the energies change accordingly. As the oxyhemoglobin molecular box shrinks, the possible energies of the oxygen is increased. For large quantum numbers(needed for reasonable energies at macroscopic dimensions) the spacing between nodes in the wavefunctions becomes too small to measure. The result is that the oxygen has an equal probability of being anywhere in the oxyhemoglobin molecular box. Similarly, the relative difference in energy between levels becomes too small to measure, so the energy the oxygen can have appears continuous rather than quantized when the quantum numbers become large. Both position and energy can be described by classical mechanics at large quantum numbers.

## Funding

The Federal University of Technology Minna, Nigeria supports the research activities.

